# Reducing nihilism in metastatic pancreatic ductal adenocarcinoma: Treatment, sequencing, and effects on survival outcomes

**DOI:** 10.1002/cam4.3477

**Published:** 2020-09-30

**Authors:** Eileen M. O'Reilly, Paul Cockrum, Andy Surinach, Zheng Wu, Allison Dillon, Kenneth H. Yu

**Affiliations:** ^1^ Memorial Sloan Kettering Cancer Center and Weill Cornell Medical College New York NY USA; ^2^ Ipsen Cambridge MA USA; ^3^ Genesis Research Hoboken NJ USA

**Keywords:** antineoplastic combined chemotherapy protocols, carcinoma, pancreatic ductal, electronic health records, retrospective studies, survival analysis

## Abstract

**Background:**

Real‐world practice patterns, treatment sequencing, and outcomes in patients with metastatic pancreatic cancer remain unclear. Previous research indicates that the likelihood of patients with metastatic pancreatic cancer receiving or continuing cancer‐directed therapy is low—a phenomenon called nihilism. This retrospective, descriptive analysis examined clinical characteristics, treatment patterns, and outcomes for patients with metastatic pancreatic ductal adenocarcinoma (mPDAC).

**Methods:**

Treatment patterns were examined using electronic health records from the Flatiron Health database covering the period from January 1, 2014, to June 30, 2019. Real‐world overall survival [rwOS]) was compared for a subgroup of patients receiving treatment and a matched subgroup not receiving treatment.

**Results:**

Of 7666 patients, 5687 (74.2%) received at least one line of systemic therapy. A greater proportion of patients receiving treatment than not receiving treatment had an initial diagnosis of stage IV disease (68.8% vs 61.2%, respectively). Among patients receiving an initial therapy, fewer than half (38.2%; 2174/5687) received second‐line treatment, mostly because they died, and only 34.3% (745/2174) of those receiving second‐line treatment advanced to third‐line treatment. The rwOS for patients receiving at least one line of systemic therapy was 8.1 months versus 2.6 months for matched patients not receiving treatment (hazard ratio, 0.41; 95% confidence interval, 0.38‐0.45; 1470 patients per group).

**Conclusions:**

Systemic therapy provided significant clinical benefit for patients who were eligible and chose to receive it, particularly when treatment was consistent with guideline recommendations. The large proportion of patients initiating treatment suggests that nihilism with mPDAC is diminishing.

## Graphical abstract

In metastatic pancreatic ductal adenocarcinoma, systemic therapy provided clinical benefit for patients who were eligible and chose to receive it, particularly when treatment was consistent with guideline recommendations. The large proportion of patients initiating treatment in this study suggests that nihilism with metastatic pancreatic ductal adenocarcinoma is diminishing.

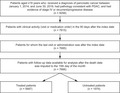

**LAY SUMMARY**

- Newly diagnosed pancreatic cancer has often already spread (metastasized) and cannot be cured surgically.
- Patients do not always receive medical (nonsurgical) treatments because of a perceived lack of value (nihilism). Previous studies with medical‐therapy combinations, however, showed survival benefits in metastatic pancreatic ductal adenocarcinoma (mPDAC; the most common type of pancreatic cancer).
- The authors examined medical records for 7666 patients with mPDAC in the USA. The proportion receiving medical treatment was higher than reported previously for similar patients, and there was an observed survival benefit.
- The data suggest diminishing nihilism in pancreatic cancer and highlight the benefit of therapy.

## 1 INTRODUCTION

It has been estimated that pancreatic cancer will account for 3% of new cancer diagnoses in the USA in 2020, corresponding to 57,600 new cases.^1^ It is one of the most lethal cancers and is predicted to become the third leading cause of cancer‐related death in 2020, with 47,050 deaths anticipated.^1^ Compared with other cancers, there has been very little improvement in survival rates for patients with pancreatic cancer over the past 40 years.^1^ A 5‐year survival rate of 10% was recently reported by the American Cancer Society;^1^ although this is an increase from 4% in 1987–1989, it is, nonetheless, still the lowest survival rate of any cancer in the report,^1^ and it has been estimated that pancreatic cancer will become the second leading cause of cancer‐related death in the USA by 2030.^2^

Pancreatic ductal adenocarcinoma (PDAC), the most common type of pancreatic cancer,^3^ is difficult to diagnose and treat, in part because it is characterized by early dissemination and aggressive biology. The overexpression of immunosuppressive cytokines, the inactivity of tumor suppressor oncogenes, and a strong immunosuppressive microenvironment facilitate tumor growth, invasion, and metastasis.^4^, ^5^ Additionally, the dense stroma that surrounds PDACs may inhibit drugs from reaching the tumor, further facilitating tumor survival and growth.^6^ Pancreatic cancer is also generally difficult to diagnose because signs and symptoms, such as pain and weight loss, are largely nonspecific.^7^ Resection, meanwhile, is the only potentially curative intervention, but delays in diagnosis mean that fewer than 20% of patients have resectable disease at diagnosis.^1^ The survival rate, moreover, for advanced disease is especially low (3%).^1^

For over 20 years, gemcitabine alone was the only treatment approved for metastatic PDAC (mPDAC). In the past 10 years, however, two chemotherapeutic drug combinations, 5‐fluorouracil (5‐FU) + leucovorin (LV) + non‐liposomal irinotecan + oxaliplatin (FOLFIRINOX) and gemcitabine + nanoparticle albumin‐bound paclitaxel (gem/nab; ABRAXANE, Celgene Corporation), have both demonstrated improvements in overall survival (OS) relative to gemcitabine alone when used as first‐line treatments.^8^, ^9^ In 2015–2016, liposomal irinotecan (ONIVYDE [historical names include nal‐IRI, MM‐398, or PEP02], Ipsen Biopharmaceuticals, Inc.) was approved for use in combination with 5‐FU/LV for the treatment of mPDAC following progression with gemcitabine‐based therapy.^10^, ^11^ This combination was shown to improve OS compared with 5‐FU/LV alone^12^ and is currently the only post‐gemcitabine regimen with a category 1 National Comprehensive Cancer Network® (NCCN) recommendation.^13^ Given these recent developments, major clinical practice guidelines now generally include recommendations for FOLFIRINOX or gem/nab as first‐line therapy^3^, ^13^, ^14^ and gem/nab or liposomal irinotecan +5‐FU/LV as second‐line therapy (after progression with defined first‐line therapies) in patients meeting specific additional criteria, including good performance status.^3^, ^13^, ^14^ Despite these therapeutic advances and associated updates to clinical practice guidelines, the nihilism associated with the management of patients with mPDAC that has been evident for over several decades^15^, ^16^ continues among patients and the physicians treating them. Indeed, studies have shown that patients with a diagnosis of noncurative PDAC or stage IV pancreatic cancer often do not receive cancer‐directed treatment.^17^, ^18^ Moreover, treatment recommendations for patients with poor performance status are more limited; there are no recommended therapies after second line, regardless of performance status.^3^, ^13^, ^14^ Nihilism surrounding the prognosis for patients with mPDAC thus continues to limit important discussions about available treatment options.^16^

Real‐world evidence supports decision makers who assess therapeutic and economic options for managing patients, including those not eligible for clinical trials who receive standard care. The goal of this analysis was to supplement evidence from clinical trials by evaluating practice patterns, treatment sequencing, and outcomes in patients with mPDAC treated in a real‐world setting since 2014.

## 2 METHODS

### 2.1 Study design and data source

This retrospective, descriptive analysis was performed with data from Flatiron Health, a demographically and geographically diverse US database of electronic‐health‐record information from over 280 cancer clinics,^19^ representing more than 2.2 million patients with cancer. The data include structured and unstructured patient‐level information, curated via technology‐enabled abstraction. Structured data elements include patient demographics and treatments; unstructured elements include pathology, physician and discharge notes, and radiology reports. Data were deidentified, with provisions to prevent re‐identification, which would violate patient privacy and data confidentiality.

### 2.2 Study population

This study included patients aged 18 years or older who were considered to have mPDAC based on: a diagnosis of pancreatic cancer (codes 157.xx and code C25.xx, respectively, from the International Classification of Diseases and Related Health Problems, 9th and 10th revisions, Clinical Modification) between January 1, 2014, and June 30, 2019; pathology consistent with PDAC; and evidence of stage IV or progressive/recurrent disease. The index date for each patient was defined as the date they received a metastatic diagnosis (for the purposes of the study, this was also considered to be the date of initial diagnosis). Patients were also required to have at least two documented clinical visits, with one during the 90 days after the index date to ensure patient continuity in the database. Patients with no follow‐up data in the database were excluded.

### 2.3 Treatment initiation and sequencing

A series of operational definitions informed by clinical input were used to categorize treatments received after, or up to 14 days before, the index date into therapy lines. All drugs administered in the 28 days after an initial therapy were considered part of the same regimen. The addition of a new therapy after 28 days was considered the start of a new regimen unless, in the 90 days after the start of the treatment line: 5‐FU was substituted for capecitabine or vice versa; LV was substituted for levoleucovorin or vice versa; LV/levoleucovorin was added; or protein‐bound paclitaxel was added to a gemcitabine regimen or vice versa. Regimens were grouped according to the agents they contained, irrespective of loading or maintenance doses and frequency of administration. Based on these definitions, lines of therapy in the study may differ from those received in clinical practice. For example, treatment received in an adjuvant/neoadjuvant setting before metastatic diagnosis may not have been recorded as part of this study. Chemoradiation treatment was not included in the analyses.

### 2.4 Data collection and study outcomes

Baseline demographic and clinical characteristics included age, sex, race, smoking history, geographic region, year of diagnosis, year of treatment, tumor location and stage, Eastern Cooperative Oncology Group (ECOG) performance status score, serum albumin level, and practice type. The primary endpoint was real‐world OS (rwOS) from the date of metastatic diagnosis or from the start of each line of therapy. Length of therapy was defined as the number of days between the date of initiation and the last date of the last treatment cycle; no censoring was employed.

### 2.5 Statistical analyses

Baseline demographic and clinical characteristics were analyzed descriptively. A greedy nearest‐neighbor‐matching algorithm utilizing the propensity score matched each control unit (patient not receiving treatment) with each unit in the case group (patient receiving treatment) such that matching produced the smallest within‐pair difference among all available pairs with this case unit. The propensity score was estimated using age at index date, sex, race, smoking history, tumor stage at initial diagnosis, year of metastatic diagnosis, and practice type. Exact matches were required for age at index date, sex, tumor stage, and year of metastatic diagnosis. Patients were matched if the difference in the logits of the propensity score was no more than 0.2 times the pooled estimate of the common standard deviation of the logits of the propensity scores. Differences in baseline characteristics between treated and untreated patients in this matched cohort were assessed with Pearson chi‐squared tests (categorical variables) and *t*‐tests (continuous variables).

Kaplan–Meier methods were used to estimate median rwOS and survival probabilities. For the survival analyses, death dates were imputed to the 15th day of the month. Data for patients who did not die were censored at their last recorded, structured activity. The differences in rwOS between treated and untreated patients were assessed using a Cox proportional hazards model, with *P* values derived from a log‐rank test. A *p*‐value <.05 was considered statistically significant.

Treatment patterns and sequencing were assessed for unmatched, treated patients. The frequency and proportion of patients receiving one, two, or three lines of therapy were calculated. Treatment regimens were examined by line and year of initiation, and treatment sequences (first line only; first and second lines; and first, second, and third lines) were constructed. For analysis purposes, the term “regimen” was applied to any systemic treatment that contained at least one chemotherapeutic agent.

Statistical analyses were performed using SAS statistical software, version 9.4 (SAS Institute Inc.).

## 3 RESULTS

### 3.1 Patients

**FIGURE 1:**
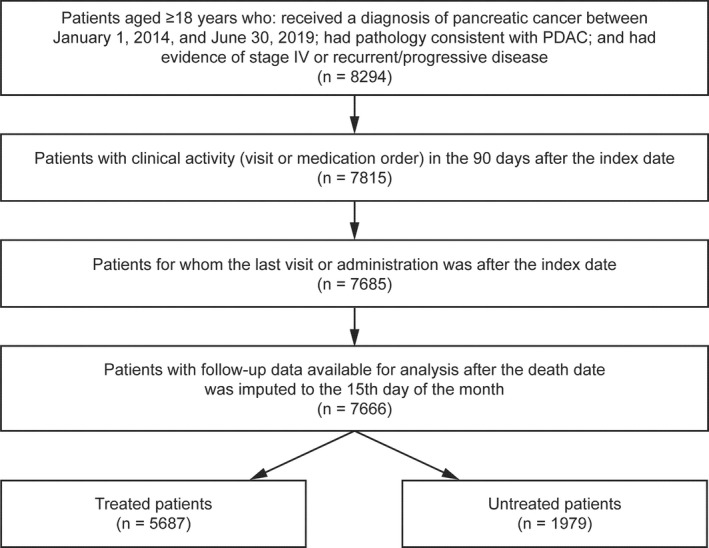
Patients meeting study inclusion criteria. Abbreviations: PDAC, pancreatic ductal adenocarcinoma

Of 7666 patients with mPDAC who had data available for analysis, 5687 (74.2%) received at least one line of systemic therapy (Figure 1). Unadjusted and propensity‐matched patient demographic and clinical characteristics for treated and untreated groups are shown in Table 1. In the unmatched cohort, treated patients, versus untreated patients, were as follows: slightly younger (median age, 68 years vs 71 years, respectively) at the index date (metastatic diagnosis); more likely to receive an initial diagnosis of stage IV disease (68.8% vs 61.2%, respectively); and less likely to receive care at academic centers (12.6% vs 20.9%, respectively). Propensity score matching yielded treated and untreated groups of 1470 patients each (Table 1). Despite matching, there were small but statistically significant differences between treated and untreated patients in smoking history, ECOG performance status score, and serum albumin level.

**TABLE 1:** Demographic and clinical characteristics for treated and untreated patients

	All Patients (n = 7666)	Unmatched Cohort		Matched Cohort^a^		
		Treated Patients (n = 5687)	Untreated Patients (n = 1979)	Treated Patients (n = 1470)	Untreated Patients (n = 1470)	*P* value^b^
Age at index date,^c^ y						1
Mean (SD)	68.1 (9.7)	67.4 (9.7)	70.1 (9.5)	70.7 (8.6)	70.7 (8.6)	
Median (IQR)	69.0 (62.0‐76.0)	68.0 (61.0‐75.0)	71.0 (64.0‐78.0)	71.0 (65.0‐78.0)	71.0 (65.0‐78.0)	
BMI, kg/m^2^, mean (SD)^d^	26.0 (7.5)	26.1 (6.2)	25.8 (10.1)	26.2 (7.8)	26.1 (11.5)	.908
Men, no. (%)	4106 (53.6)	3082 (54.2)	1024 (51.7)	758 (51.6)	758 (51.6)	1
Race, no. (%)						
Asian	128 (1.7)	94 (1.7)	34 (1.7)	21 (1.4)	24 (1.6)	.900
White	5368 (70.0)	4022 (70.7)	1346 (68.0)	1050 (71.4)	1050 (71.4)	
Other or missing	2170 (28.3)	1571 (27.6)	599 (30.3)	399 (27.1)	396 (26.9)	
Smoking history, no. (%)						<.0001
History of smoking	4325 (56.4)	3234 (56.9)	1091 (55.1)	459 (31.2)	811 (55.2)	
No history of smoking	3308 (43.2)	2434 (42.8)	874 (44.2)	1007 (68.5)	654 (44.5)	
Unknown or not documented	33 (0.4)	19 (0.3)	14 (0.7)	4 (0.3)	5 (0.3)	
Tumor location in pancreas, no. (%)						.632
Head	3812 (49.7)	2793 (49.1)	1019 (51.5)	735 (50.0)	722 (49.1)	
Other	3854 (50.3)	2894 (50.9)	960 (48.5)	735 (50.0)	748 (50.9)	
Tumor stage at initial diagnosis, no. (%)						1
Stage IV	5122 (66.8)	3910 (68.8)	1212 (61.2)	985 (67.0)	985 (67.0)	
Other	2544 (33.2)	1777 (31.2)	767 (38.8)	485 (33.0)	485 (33.0)	
Year of metastatic diagnosis, no. (%)						1
2014	1233 (16.1)	916 (16.1)	317 (16.0)	246 (16.7)	246 (16.7)	
2015	1398 (18.2)	1028 (18.1)	370 (18.7)	273 (18.6)	273 (18.6)	
2016	1413 (18.4)	1040 (18.3)	373 (18.8)	287 (19.5)	287 (19.5)	
2017	1533 (20.0)	1166 (20.5)	367 (18.5)	286 (19.5)	286 (19.5)	
2018	1545 (20.2)	1154 (20.3)	391 (19.8)	293 (19.9)	293 (19.9)	
2019	544 (7.1)	383 (6.7)	161 (8.1)	85 (5.8)	85 (5.8)	
Surgery before metastatic diagnosis, no. (%)	1398 (18.2)	1000 (17.6)	398 (20.1)	262 (17.8)	257 (17.5)	.809
Time from surgery to metastatic diagnosis, months						.487
Mean (SD)	16.1 (16.3)	15.7 (15.5)	17.1 (18.1)	16.5 (17.5)	15.5 (15.1)	
Median (IQR)	11.6 (6.2‐19.3)	11.6 (6.1‐19.2)	11.4 (6.5‐20.2)	12.6 (5.7‐20.1)	11.0 (5.9‐18.2)	
Geographic region, no. (%)						.505
Northeast	1235 (16.1)	915 (16.1)	320 (16.2)	253 (17.2)	263 (17.9)	
Midwest	906 (11.8)	701 (12.3)	205 (10.4)	186 (12.7)	172 (11.7)	
South	3163 (41.3)	2439 (42.9)	724 (36.6)	629 (42.8)	603 (41.0)	
West	1065 (13.9)	805 (14.2)	260 (13.1)	195 (13.3)	204 (13.9)	
Puerto Rico	64 (0.8)	52 (0.9)	12 (0.6)	13 (0.9)	8 (0.5)	
Unknown	1233 (16.1)	775 (13.6)	458 (23.1)	194 (13.2)	220 (15.0)	
ECOG performance status score, no. (%)^d^						.004
0	628 (8.2)	502 (8.8)	126 (6.4)	127 (8.6)	97 (6.6)	
1	827 (10.8)	617 (10.8)	210 (10.6)	152 (10.3)	164 (11.2)	
≥2	303 (4.0)	179 (3.1)	124 (6.3)	55 (3.7)	91 (6.2)	
Missing	5908 (77.1)	4389 (77.2)	1519 (76.8)	1136 (77.3)	1118 (76.1)	
Serum albumin, g/dL, no. (%)^d^						.001
<4	1657 (21.6)	1128 (19.8)	529 (26.7)	310 (21.1)	379 (25.8)	
≥4	1006 (13.1)	802 (14.1)	204 (10.3)	192 (13.1)	145 (9.9)	
Unknown	5003 (65.3)	3757 (66.1)	1246 (63.0)	968 (65.9)	946 (64.4)	
Practice type, no. (%)						1
Academic	1130 (14.7)	716 (12.6)	414 (20.9)	183 (12.4)	183 (12.4)	
Community	6536 (85.3)	4971 (87.4)	1565 (79.1)	1287 (87.6)	1287 (87.6)	

Abbreviations: BMI, body mass index; ECOG, Eastern Cooperative Oncology Group; IQR, interquartile range; SD, standard deviation.

^a^ Patients were matched exactly for age at index date, sex, year of metastatic diagnosis, and tumor stage at initial diagnosis; the propensity score was estimated using these variables, as well as race, smoking history, and practice type.

^b^ Differences between treated and untreated patients in the matched cohort were assessed with Pearson chi‐squared tests (categorical variables) and *t*‐tests (continuous variables).

^c^ In the Flatiron Health database, the recording of patient age is capped at 85 years to protect patient confidentiality.

^d^ The closest record that occurred within 30 days prior to or on the metastatic diagnosis date was reported.

### 3.2 Treated versus untreated patients

**FIGURE 2:**
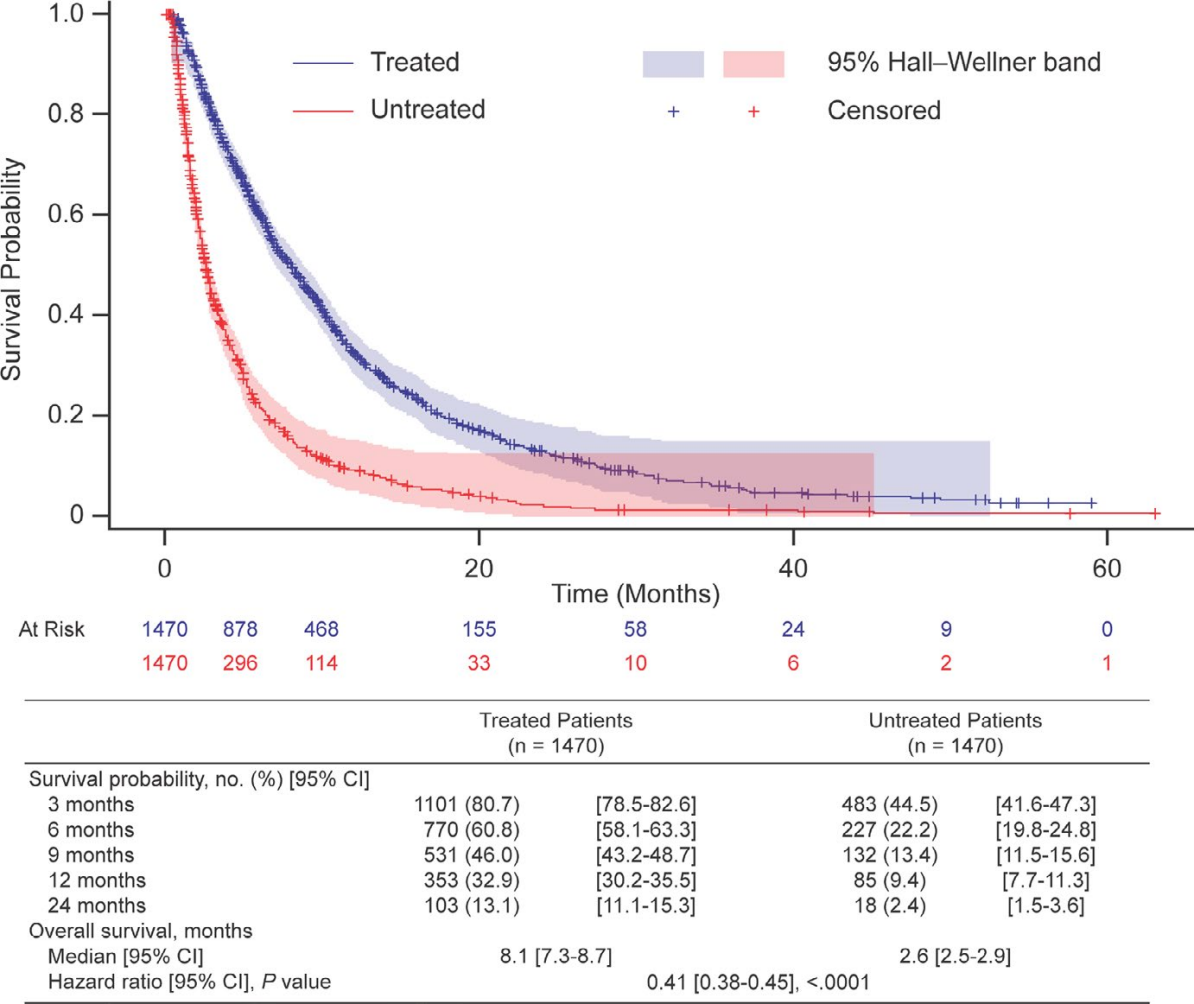
Overall survival and survival milestones for treated and untreated patients (matched cohort). Survival is measured from the date of metastatic diagnosis. Death dates were imputed to the 15th day of the month or, for patients who did not die during the study, data were censored at their last recorded, structured activity. The hazard ratio is from a Cox proportional hazards model, and the *p* value is from a log‐rank test. Abbreviations: CI, confidence interval

Observed differences in rwOS and survival probabilities at key milestones between treated and untreated patients were similar for unmatched (data not shown) and matched cohorts. In the matched cohort, patients who received treatment had a significantly longer median rwOS since the metastatic diagnosis than patients not receiving treatment (8.1 months vs 2.6 months, respectively; hazard ratio, 0.41; 95% confidence interval [CI], 0.38‐0.45; *p* < .0001; Figure 2). Survival probabilities across milestones in the matched cohort were twofold to fivefold higher for patients receiving treatment than for those not receiving treatment (Figure 2).

### 3.3 Treatment patterns and sequencing analysis

In total, 5687 patients in the unmatched cohort received initial therapy. Database records do not indicate whether patients completed the therapy, but a 46.2% (2625/5687) died during or after first‐line therapy and 38.2% (2174/5687) received second‐line therapy. Only 745 patients (34.3%) who received second‐line therapy advanced to third‐line therapy (1129/2174, 51.9%, died during or after receiving second‐line therapy). Overall, 13.1% of patients initially treated upon metastatic diagnosis received third‐line therapy. Across all regimens, median rwOS from the start of each therapy line was highest for first‐line and lowest for third‐line therapy (first‐line, 7.1 months [95% CI, 6.8‐7.3], 5687 patients; second‐line, 5.6 months [5.3‐6.0], 2166 patients; third‐line, 4.3 months [4.0‐4.7], 742 patients).

**FIGURE 3:**
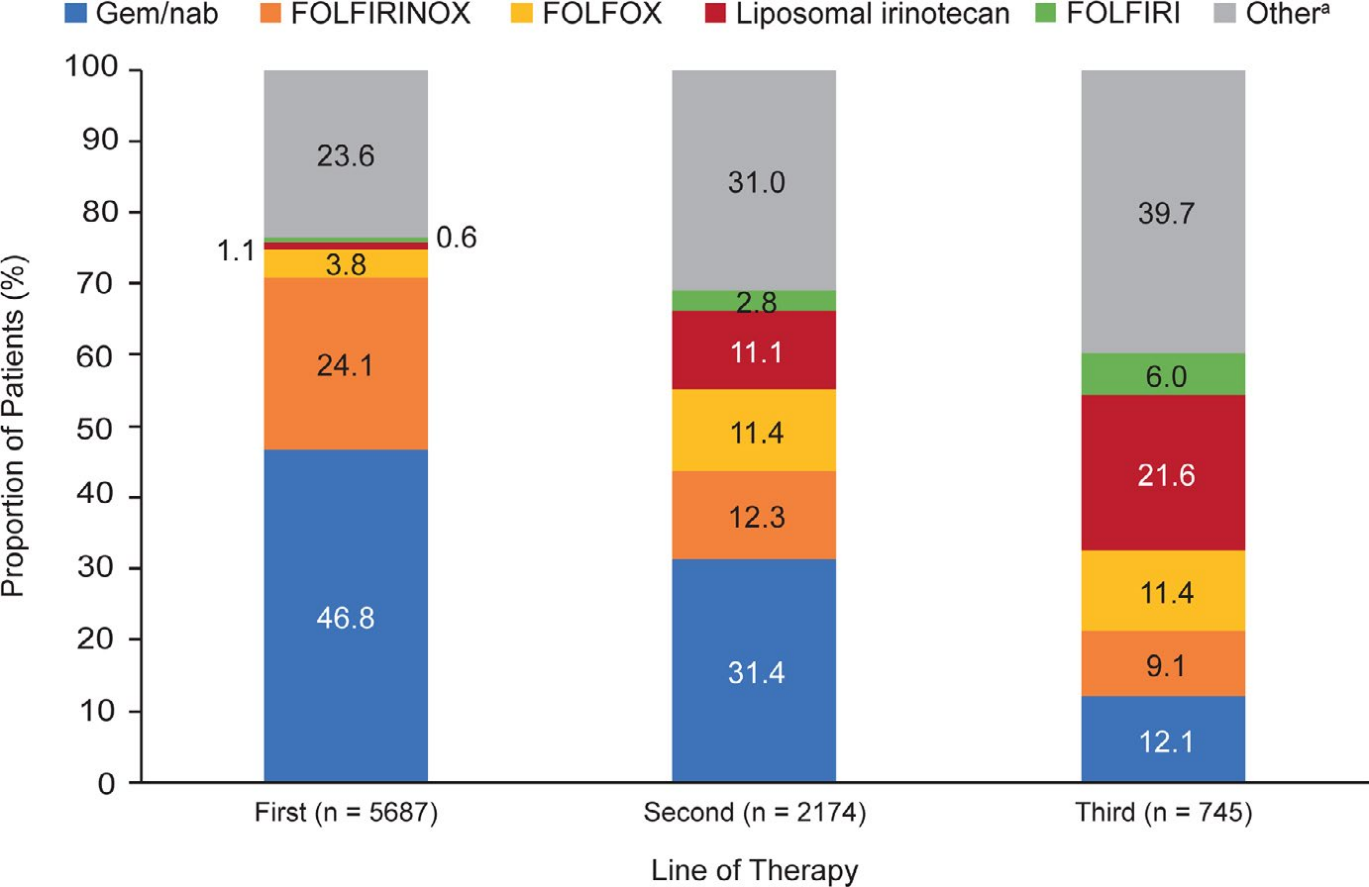
Treatment regimen use by line of therapy (unmatched cohort). For reporting purposes, regimens representing ≤1% of patients per line of therapy were combined as “other” regimens, except for FOLFIRINOX, FOLFOX, and FOLFIRI. Abbreviations: FOLFIRI, 5‐fluorouracil + leucovorin + non‐liposomal irinotecan; FOLFIRINOX, 5‐fluorouracil + leucovorin + non‐liposomal irinotecan + oxaliplatin; FOLFOX, 5‐fluorouracil + leucovorin + oxaliplatin; gem/nab, gemcitabine + nanoparticle albumin‐bound paclitaxel.^a^Includes any unique, single chemotherapeutic agent or combination of agents other than those listed by name or acronym above. A full list of regimens is available in the Supporting Information

Many unique regimens were reported (142, 141, and 117 as first‐, second‐, and third‐line therapies, respectively). For reporting purposes, regimens representing up to 1% of patients per therapy line were combined as “other” regimens, except for FOLFIRINOX, 5‐FU/LV +oxaliplatin (FOLFOX), and 5‐FU/LV +non‐liposomal irinotecan (FOLFIRI). The most common first‐line therapy was gem/nab, followed by FOLFIRINOX (Figure 3). Gem/nab and FOLFIRINOX were less commonly used as second‐ and third‐line therapies than as first‐line therapies, but the reverse was true for liposomal irinotecan, FOLFOX, and “other” regimens. Liposomal irinotecan, gem/nab, and FOLFOX were the most frequently used third‐line therapies. A full list of regimens is available in the Supporting Information.

**FIGURE 4:**
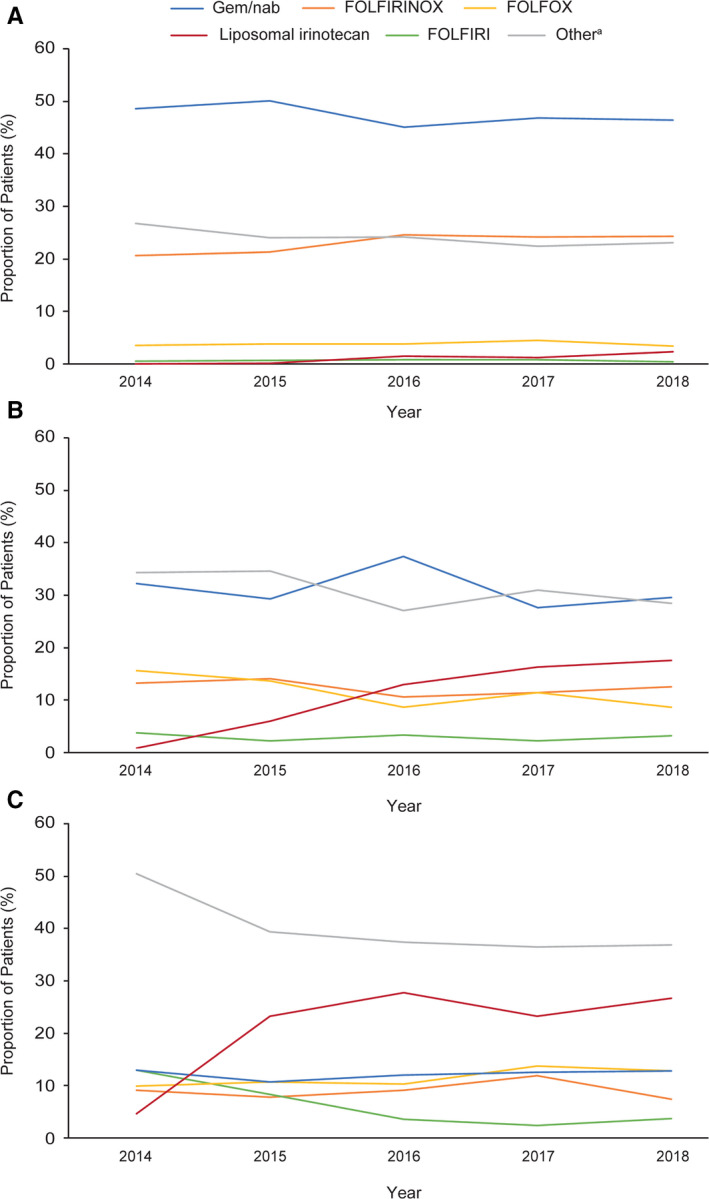
Use of common (A) first‐line, (B) second‐line, and (C) third‐line regimens by year. Abbreviations: FOLFIRI, 5‐fluorouracil + leucovorin + non‐liposomal irinotecan; FOLFIRINOX, 5‐fluorouracil + leucovorin + non‐liposomal irinotecan + oxaliplatin; FOLFOX, 5‐fluorouracil +leucovorin + oxaliplatin; gem/nab, gemcitabine + nanoparticle albumin‐bound paclitaxel.^a^Includes any unique, single chemotherapeutic agent or combination of agents other than those listed by name or acronym above. A full list of regimens is available in the Supporting Information

Figure 4 shows the percentage of patients receiving frequently used regimens by therapy line for 2014–2018. The most common first‐line treatment was gem/nab, followed by FOLFIRINOX. FOLFIRINOX use increased from 20.6% in 2014 to 24.3% in 2018, whereas gem/nab use decreased from 50.1% (highest value) in 2015 to 46.4% in 2018; small decreases were also recorded for “other” regimens. FOLFIRINOX and gem/nab use as second‐line therapies decreased over time (from 13.3% to 12.5% and from 32.3% to 29.6%, respectively); in contrast, after the introduction of liposomal irinotecan in the USA in 2015, its use as second‐line therapy increased to 13.0% in 2016 and reached 17.6% in 2018. FOLFOX, FOLFIRI, and “other” regimens represented a greater proportion of second‐line than first‐line treatments. FOLFIRINOX and gem/nab were used less as third‐line than as second‐line therapies, whereas the reverse was true for liposomal irinotecan (23.2% at its lowest and 27.7% at its highest for third line between 2016 and 2018) and “other” regimens (50.4% in 2014 and 36.7% in 2018 for third line).

Table 2 reports the most frequent treatment sequences and associated median rwOS values from the date of metastatic diagnosis. Most patients received only one regimen, most commonly gem/nab, but median rwOS values were higher for two‐ and three‐line therapies than for one‐line therapies. The longest rwOS was with FOLFIRINOX followed by gem/nab (or vice versa) then liposomal irinotecan +5‐FU/LV. A full list of treatment sequences is available in the Supporting Information.

**TABLE 2:** Real‐World Overall Survival for Common Treatment Sequences

Sequence	Patients, no. (%)^a^	Median (95% CI) rwOS,^b^ months
One‐line sequences		
Gem/nab	1752 (30.8)	5.1 (4.7‐5.4)
FOLFIRINOX	712 (12.5)	5.8 (5.4‐6.4)
Gem monotherapy	389 (6.8)	3.5 (3.2‐4.0)
FOLFOX	134 (2.4)	3.4 (3.0‐4.6)
Two‐line sequences		
FOLFIRINOX >> gem/nab	346 (6.1)	11.0 (10.5‐12.0)
Gem/nab >> FOLFIRINOX	132 (2.3)	10.3 (9.0‐11.3)
Gem/nab >> liposomal irinotecan +5‐FU/LV	122 (2.1)	11.5 (10.5‐12.7)
Gem/nab >> FOLFOX	114 (2.0)	10.3 (9.1‐11.6)
Gem/nab >> capecitabine	54 (0.9)	9.9 (8.5‐12.5)
Three‐line sequences		
FOLFIRINOX >> gem/nab >> liposomal irinotecan +5‐FU/LV	36 (0.6)	17.7 (12.1‐19.5)
Gem/nab >> FOLFIRINOX >> liposomal irinotecan +5‐FU/LV	14 (0.2)	13.7 (7.6‐21.8)

Abbreviations: >>, therapy advanced from one line to another; 5‐FU, 5‐fluorouracil; CI, confidence interval; FOLFIRINOX, 5‐fluorouracil + leucovorin + non‐liposomal irinotecan + oxaliplatin; FOLFOX, 5‐fluorouracil + leucovorin + oxaliplatin; gem/nab, gemcitabine + nanoparticle albumin‐bound paclitaxel; LV, leucovorin; rwOS, real‐world overall survival.

^a^ Reflects the number of patients who advanced through one, two, or three lines of therapy, receiving a particular one‐, two‐, or three‐line sequence; expressed as a percentage of all patients who received at least one line of therapy (n = 5687).

^b^ rwOS is measured from the start of each line of therapy

## 4 DISCUSSION

In the past decade, there have been several advances in the treatment of patients with mPDAC, including the publication of high‐quality evidence supporting the use of multi‐agent, combination cytotoxic regimens as first‐ and second‐line treatments.^8^, ^9^, ^12^ The large, retrospective analysis presented here, which was based on geographically diverse electronic‐health‐record data for patients with mPDAC in the USA, is the first to provide a contemporary description of real‐world treatment patterns and associated outcomes, and suggests that the disease management is evolving in response to these advances.

The proportion of patients with PDAC receiving systemic chemotherapy in the metastatic setting (74.2%) in the present analysis is higher than those reported from patient databases in previous studies for stage IV pancreatic cancer^18^, ^20^ and noncurative PDAC.^17^ In total, 140,210 patients with stage IV pancreatic cancer were included in the National Cancer Data Base from 2000 to 2011; the proportion receiving systemic treatment increased from 47% to 52% during the study.^18^ Of 10,881 patients in the Ontario Cancer Registry who received a new diagnosis of noncurative PDAC between 2005 and 2016, 38.1% received cancer‐directed therapy (including chemotherapy [26.6%] and chemoradiation therapy [11.5%]).^17^ Among 12, 978 Medicare Advantage enrollees diagnosed with PDAC between January 2011 and May 2017 only 43% of patients received chemotherapy.^20^ No trends over time were reported. Comparing these studies with the current analysis suggests that a general increase in cancer‐directed therapy has occurred recently. This increase may reflect the fact that previous studies^17^, ^18^ were conducted, in whole or in part, before the approval of liposomal irinotecan +5‐FU/LV for the treatment of mPDAC following progression with gemcitabine‐based therapy and before the publication of data demonstrating the efficacy of FOLFIRINOX and gem/nab as first‐line mPDAC treatments.^8^, ^9^

In the current analysis, patients who received treatment had a median rwOS from the date of metastatic diagnosis that was just over 5 months longer than that of matched untreated patients, a reduction of approximately 60% in the risk of death for treated patients. This suggests that evidence supporting new therapeutic options has led to increases in the number of patients treated at the time of metastatic diagnosis and, most importantly, increases in survival. This is supported by recent US population analyses of the trends in 5‐year relative survival among patients with mPDAC which has increased from 3.7% to 5.1% between 2002 and 2010.^21^

In this study, the real‐world treatment patterns in mPDAC were consistent with recommendations from the NCCN, the American Society of Clinical Oncology and the European Society for Medical Oncology.^3^, ^13^, ^14^ Although many unique regimens were received by small numbers of patients, the majority received first‐line treatment with gem/nab or FOLFIRINOX, then whichever of these two they had not already received if advancing to second‐line therapy. The percent of patients who received third‐line therapy in this study (34.3%) is higher than previously reported in a sample of 4011 patients with mPDAC treated in U.S. oncology practices (17%).^22^ The most common third‐line regimens included liposomal irinotecan following first‐ or second‐line gemcitabine‐based treatment.

Owing to the difficulty in conducting randomized studies in metastatic pancreatic cancer in terms of feasibility and clinical success, there are few randomized clinical studies in patients with mPDAC receiving second‐line therapy and none recently reported in those receiving third‐line therapy. Definitive evidence to inform treatment‐sequencing strategies is, thus, currently limited, and clinical decision‐making relies on real‐world evidence, which is mostly retrospective. The rwOS data from the present analysis are, thus, a valuable addition to the evidence base, even though a full comparative assessment was precluded by small numbers of patients receiving some sequences. The most commonly used two‐line sequences were generally associated with similar rwOS values. Samples sizes were too small to support comparisons among many three‐line sequences, but those involving liposomal irinotecan +5‐FU/LV following gem/nab and FOLFIRINOX had higher rwOS values than the common second‐line sequences.

### 4.1 Limitations

Several limitations inherent in the analyses of real‐world data should be considered. Nonrandom allocation and bias in the frequency and availability of data (e.g., systematic differences in terms of missing data or data‐collection frequency) are general limitations and cannot be controlled completely with statistical methods. Propensity score matching was used to control for patient‐level factors known or hypothesized to be associated with treatment. Although a good balance was achieved across most variables, unobserved differences may remain, which could have influenced the results. We also acknowledge that ECOG performance status scores at the time of metastatic diagnosis were missing for approximately 75% of patients and that organ dysfunction could not be extracted from the data; both variables may have influenced the treatments initiated and selected and the rwOS observed. The recording of patient age is capped at 85 years in the database to protect patient confidentiality; the true age of some elderly patients with mPDAC and associated clinical outcomes could not, therefore, be determined. rwOS for some regimens by line and/or year of therapy was limited by small sample sizes. The requirement of at least two documented clinical visits may have biased the cohort to patients who were able to continue to seek care and thus have better overall survival outcomes than the general mPDAC population. Finally, these results may not be generalizable outside community oncology clinics.

## 5 CONCLUSIONS

Important observations regarding practice patterns and treatment sequencing may be provided by research based on real‐world data. In this study of 7666 patients who received a diagnosis of mPDAC, 74.2% received systemic therapy, representing a significant increase from previous reports in similar patients and suggesting that nihilism in metastatic pancreatic cancer is diminishing. Although few patients advanced to later lines of therapy (only 13.1% of patients who were initially treated upon metastatic diagnosis received third‐line therapy), the most common sequences received were generally consistent with clinical practice guidelines and, overall, median rwOS for patients receiving first‐line treatment was 8.1 months from the date of metastatic diagnosis (compared with 2.6 months for patients who did not receive treatment).

## CONFLICTS OF INTEREST

Eileen M. O’Reilly's institution has received research funding from Arcus, AstraZeneca, BioAtla, BioNTech, Bristol‐Myers Squibb, Celgene, Genentech, Roche and Silenseed, and she has received compensation for consulting and advisory services from BioLineRx, Bristol‐Myers Squibb, CytomX Therapeutics, Ipsen, Merck, Polaris, Rafael and Targovax.; Paul Cockrum is an employee of Ipsen and holds stock or stock options; Andy Surinach, Zheng Wu, and Alison Dillon are employees of Genesis Research, which receives consulting fees from Ipsen; Kenneth H. Yu has received research funding from Bristol‐Myers Squibb and Halozyme, and compensation for advisory services from Ipsen.

## AUTHORS’ CONTRIBUTIONS

Eileen M. O’Reilly: conceptualization, methodology, visualization, and writing—review and editing. Paul Cockrum: conceptualization, formal analysis, funding acquisition, investigation, methodology, project administration, resources, visualization, writing—original draft, and writing—review and editing. Andy Surinach: conceptualization, formal analysis, investigation, methodology, project administration, resources, visualization, and writing—review and editing. Zheng Wu: formal analysis, investigation, methodology, and visualization. Alison Dillon: formal analysis, investigation, methodology, and visualization. Kenneth H. Yu: conceptualization, methodology, visualization, and writing—review and editing.

## PREVIOUS PRESENTATIONS

Portions of these analyses were presented as an abstract and poster at the American Society of Clinical Oncology Gastrointestinal Cancers Symposium (ASCO GI) 2020, January 23–25, 2020, San Francisco, California.

## Supporting information

Supplementary Material

## Data Availability

Data for these analyses were made available to the authors through a third‐party license from Flatiron Health, an oncology data provider in the United States. The authors cannot make these data publicly available due to data use agreement. Researchers may access these data by licensing the metastatic pancreatic cancer data mart from Flatiron Health (https://flatiron.com/real‐world‐evidence/).
